# Epicardial Adipose Tissue Shows No Significant Incremental Value for Discriminating Coronary Artery Disease in Adults Aged 80 Years and Older: A Machine Learning Analysis

**DOI:** 10.3390/jcdd13090424

**Published:** 2026-09-01

**Authors:** Jingyi Li, Ziwei Zhu, Xuefeng Ni, Hui Lian

**Affiliations:** 1 Department of Cardiology, Peking Union Medical College Hospital, Chinese Academy of Medical Sciences and Peking Union Medical College, Beijing 100730, China; 2 Department of Health Care, Peking Union Medical College Hospital, Chinese Academy of Medical Sciences and Peking Union Medical College, Beijing 100730, China

**Keywords:** epicardial adipose tissue, coronary artery disease, very elderly adults, machine learning

## Abstract

**Background:** Epicardial adipose tissue (EAT) has been associated with coronary artery disease (CAD) in middle-aged and mixed-age populations, but its value in adults aged 80 years and older remains uncertain. This study investigated whether computed tomography (CT)-derived EAT volume provides incremental value for discriminating CAD in this very elderly population. **Methods:** We retrospectively included 155 patients aged ≥80 years who underwent coronary imaging evaluation between 2020 and 2022. EAT volume was quantified from non-contrast chest CT. Logistic regression, CatBoost modeling, and SHapley Additive exPlanations (SHAP) were used to assess the association of EAT with CAD, its discriminative value, and its relative contribution. **Results:** Forty-five patients (29.0%) had CAD. Median EAT volume did not differ between groups (*p* = 0.716) and was not associated with CAD after adjustment for age, sex, body mass index, diabetes, and glycated hemoglobin A1c (HbA1c). Adding EAT did not improve discrimination in logistic regression [area under the curve (AUC) 0.698 vs. 0.697; likelihood-ratio *p* = 0.945] or in cross-validated CatBoost analysis (AUC 0.623 vs. 0.614). SHAP ranked EAT eighth of eighteen predictors, below lipoprotein(a), total cholesterol, left atrial diameter, HbA1c, low-density lipoprotein cholesterol, diabetes, and atrial fibrillation. **Conclusions:** In adults aged 80 years and older, CT-derived EAT volume was not independently associated with CAD and added no discriminative value beyond conventional clinical risk factors. Total EAT volume may have limited utility as a CAD marker in very elderly adults.

## 1. Introduction

Epicardial adipose tissue (EAT) is the visceral fat depot of the heart, located between the myocardium and the visceral pericardium without a separating fascial plane. Physiologically, EAT supplies fatty acids to the adjacent myocardium and participates in cardiac thermoregulation. With pathological expansion, this depot shifts toward a pro-inflammatory and pro-fibrotic secretory phenotype characterized by increased secretion of tumor necrosis factor-α, interleukin-6, and leptin, which may promote endothelial dysfunction, coronary inflammation, atherosclerotic plaque formation, and adverse cardiac remodeling [1,2]. Meta-analyses and large cohort studies report greater EAT volume in patients with coronary artery disease (CAD) than in controls, with values rising in proportion to stenosis severity [3,4,5]. EAT has therefore been increasingly recognized as an imaging biomarker for CAD risk stratification, and this role has been further reinforced by deep-learning approaches that automate EAT quantification and demonstrate its independent prognostic value for major cardiovascular events beyond conventional risk factors [6].

The population aged 80 years and above is expanding faster than any other age group worldwide. In this age group, CAD accounts for a large share of hospitalization, functional decline, and death [7]. In daily practice, CAD in very elderly adults is also easily overlooked. Symptoms are often atypical or muted because of frailty, reduced physical activity, impaired symptom perception, cognitive impairment, and coexisting diseases. Instead of typical angina, patients may present with dyspnea, fatigue, functional decline, gastrointestinal discomfort, or decompensated heart failure [8,9].

Coronary computed tomography angiography and invasive coronary angiography remain the main tools for diagnosing CAD. However, in patients aged ≥80 years, their use is often limited by concerns about frailty, renal dysfunction, contrast exposure, procedural risk, and overall tolerance [10,11]. Therefore, simple and non-invasive markers that could raise suspicion of CAD before contrast-enhanced or invasive testing would be clinically useful in this population. EAT is measurable on chest CT that many of these patients have already undergone for other reasons, and automated quantification is now becoming routine [6]. Whether it adds diagnostic information beyond the clinical variables already available at this age is unclear. The evidence linking EAT to CAD comes predominantly from middle-aged cohorts and very elderly adults have either been excluded, under-represented, or analyzed together with younger patients in most previous studies. In patients over 80, traditional cardiovascular risk factors such as serum cholesterol, blood pressure, and body mass index (BMI) frequently exhibit attenuated or even inverted associations with cardiovascular outcomes, a pattern known as “reverse epidemiology,” which undermines the applicability of risk models developed in younger cohorts [12]. Aging itself profoundly alters EAT biology. In the very elderly, EAT accumulation is common in advanced age and coincides with chronic low-grade systemic inflammation, making it difficult to disentangle a pathological EAT signal from the background of age-related visceral adiposity [13,14].

Thus, we conducted a retrospective study of adults aged ≥80 years to test whether EAT volume, which is associated with CAD in middle-aged populations, retains that value at this age. A machine-learning framework incorporating post hoc interpretability analysis was used to evaluate the relative contribution of EAT alongside conventional risk factors in this age group.

## 2. Materials and Methods

### 2.1. Study Participants

This study was a retrospective analysis of clinical data collected from an older adult cohort at Peking Union Medical College Hospital. Consecutive patients aged ≥80 years who underwent coronary artery imaging evaluation between 2020 and 2022 were screened. Patients were included if their records contained coronary imaging adequate for CAD classification, EAT measurement from available imaging, transthoracic echocardiography, and relevant clinical and laboratory data. Those with incomplete imaging or missing variables necessary for analysis were excluded. After excluding patients with incomplete data, 155 patients were included in the final analysis. CAD was defined according to established clinical guidelines [15] as meeting at least one of the following: (1) ≥50% luminal stenosis in at least one major epicardial coronary artery documented by coronary imaging; (2) a documented history of myocardial infarction; or (3) prior coronary revascularization (percutaneous coronary intervention or coronary artery bypass grafting).

### 2.2. Clinical and Laboratory Data Collection

All clinical data were obtained from electronic medical records. Demographic and anthropometric variables included age, sex, waist circumference, body mass index (BMI), and blood pressure. Medical histories of hypertension, diabetes mellitus, atrial fibrillation, thyroid dysfunction, fatty liver disease and statin usage were recorded. Fasting laboratory tests included lipid profiles (total cholesterol [TC], low-density lipoprotein cholesterol [LDL-C], high-density lipoprotein cholesterol [HDL-C], triglyceride [TG], apolipoprotein A1 [ApoA1], apolipoprotein B [ApoB], lipoprotein(a) [Lp(a)] and free fatty acids [FFA]), renal markers (creatinine, blood urea nitrogen [BUN] and cystatin C), glycated hemoglobin A1c (HbA1c), high-sensitivity C-reactive protein (hsCRP), and erythrocyte sedimentation rate (ESR).

### 2.3. EAT and Echocardiographic Assessment

Non-contrast chest CT was performed on a dual-source scanner (Somatom Definition Flash, Siemens, Forchheim, Germany). Images were analyzed on a Syngo Volume workstation (Siemens) for EAT measurement. A trained radiologist, blinded to clinical data, traced the pericardial boundary from the level of the pulmonary artery bifurcation to the diaphragm to isolate the adipose tissue between the myocardium and the visceral pericardium. EAT was quantified as the total volume of voxels falling within −190 to −30 HU inside the traced region and expressed in cm^3^ [16,17].

Transthoracic echocardiography was performed according to the latest echocardiography guideline [18]. Left atrial anteroposterior diameter, right ventricular anteroposterior diameter, interventricular septal thickness, left ventricular posterior wall thickness, left ventricular end-diastolic and end-systolic diameters, left ventricular ejection fraction (LVEF), and left ventricular fractional shortening (LVFS) were recorded. Tricuspid regurgitation velocity, transmitral diastolic flow, and mitral annular tissue Doppler parameters were also obtained.

### 2.4. Statistical Analysis

All statistical analyses were performed in Python 3.12 using statsmodels, scikit-learn, CatBoost (version 1.2.1), SHAP (version 0.52.0), SciPy, pandas, and matplotlib. A two-sided *p*-value < 0.05 was considered statistically significant. Continuous variables are presented as mean ± standard deviation (SD) for normally distributed data, or as median (interquartile range, IQR) otherwise. Categorical variables are expressed as frequencies (percentages). Group comparisons were conducted using Student’s *t*-test or the Mann–Whitney U test for continuous variables, and the chi-square or Fisher’s exact test for categorical variables. The association between EAT volume and CAD was examined using logistic regression, with EAT entered as a continuous variable scaled per standard deviation. Covariates were chosen on the basis of their established relevance to CAD. As for sensitivity analysis, a further model additionally included variables that differed between groups or were considered clinically relevant (LDL-C, atrial fibrillation, left atrial diameter, and the E/A ratio). Sensitivity analyses were also performed after limiting the CAD group to angiographic stenosis only or to prior revascularization, each compared with the patients without CAD. Discrimination was quantified by the area under the receiver operating characteristic curve (AUC). To assess whether EAT added information beyond established risk factors, a clinical model was compared with and without EAT volume, using the change in AUC and a likelihood-ratio test of the nested models. To examine whether any contribution of EAT might be missed by a model assuming linear effects, the analysis was repeated with CatBoost [19]. Using the same clinical variables, model discrimination was estimated by repeated stratified five-fold cross-validation and compared with and without EAT volume. Model interpretability was assessed with SHapley Additive exPlanations (SHAP) [20], computed from a CatBoost model that incorporated the full set of clinical, laboratory, and echocardiographic variables together with EAT volume. Features were ranked by their mean absolute SHAP values to show their relative contribution to the prediction of CAD. The sample size was determined by the number of eligible patients during the study period. The minimum detectable odds ratio per 1-SD of EAT at 80% power (two-sided α = 0.05) was estimated using Hsieh’s method [21], with the variance inflation factor derived from the regression of EAT volume on the remaining covariates, and verified by simulation. For CatBoost, precision was judged from the variation in AUC across cross-validation repetitions, and events per variable are reported.

## 3. Results

### 3.1. Clinical Characteristics of the Study Population

Table 1 summarizes the characteristics of the 155 patients. The mean age was 86.5 ± 5.2 years, and 138 (89.0%) were male. Forty-five patients (29.0%) had CAD. Age, sex, and blood pressure were similar in the two groups, and BMI showed only a borderline difference (*p* = 0.052). Diabetes was more common in the CAD group (55.6% vs. 31.8%, *p* = 0.010), and HbA1c was higher (*p* = 0.002). Atrial fibrillation was also more frequent in patients with CAD (17.8% vs. 2.7%, *p* = 0.003). HsCRP and ESR were similar between groups. Total cholesterol and LDL-C were lower in the CAD group (*p* = 0.005 and 0.013), and ApoB followed the same direction (*p* = 0.053). Notably, more patients in the CAD group were on statins (66.7% vs. 48.2%, *p* = 0.055). Lp(a), by contrast, was higher in patients with CAD (*p* = 0.066). EAT volume did not differ between groups [129.75 (79.62, 163.22) vs. 117.45 (76.72, 173.25) cm^3^, *p* = 0.716]. On echocardiography, the CAD group had a larger left atrial diameter (*p* = 0.001) and a lower E/A ratio (*p* = 0.046). Left ventricular size and ejection fraction were similar in the two groups.

### 3.2. Logistic Regression Analysis of EAT and CAD

The association between EAT volume and CAD was further examined using nested multivariable logistic regression models with increasing levels of adjustment (Table 2). We found no statistical evidence of an association between EAT volume and CAD status. The adjusted odds ratio per 1-SD increase in EAT volume was 1.06 (95% CI: 0.73–1.52, *p* = 0.766) in Model 1, which was adjusted for age and sex; 0.93 (95% CI: 0.63–1.38, *p* = 0.721) in Model 2, which was additionally adjusted for BMI; and 0.98 (95% CI: 0.65–1.48, *p* = 0.930) in Model 3, which was further adjusted for diabetes and HbA1c. Among the covariates, BMI showed a borderline association with CAD in Model 2, whereas diabetes and HbA1c were not independently associated with CAD after simultaneous adjustment in Model 3. In a sensitivity analysis additionally incorporating selected variables with significant between-group differences or clinical relevance, including LDL-C, atrial fibrillation, left atrial diameter, and the E/A ratio, EAT volume again showed no significant association with CAD (OR: 0.81, 95% CI: 0.46–1.43, *p* = 0.469). EAT volume also showed no association with CAD when the CAD group was limited to angiographic stenosis only (OR: 0.78, 95% CI: 0.46–1.30, *p* = 0.340) or to prior revascularization (OR: 1.32, 95% CI: 0.72–2.41, *p* = 0.367) (Supplementary Materials). After further adjustment for statin use in Model 4, EAT volume remained not significantly associated with CAD (OR 1.00 per 1-SD, 95% CI: 0.65–1.52, *p* = 0.988). In this sample, the smallest odds ratio detectable at 80% power was 1.80 per 1-SD of EAT volume.

### 3.3. Discriminative and Incremental Predictive Value

**Figure 1:**
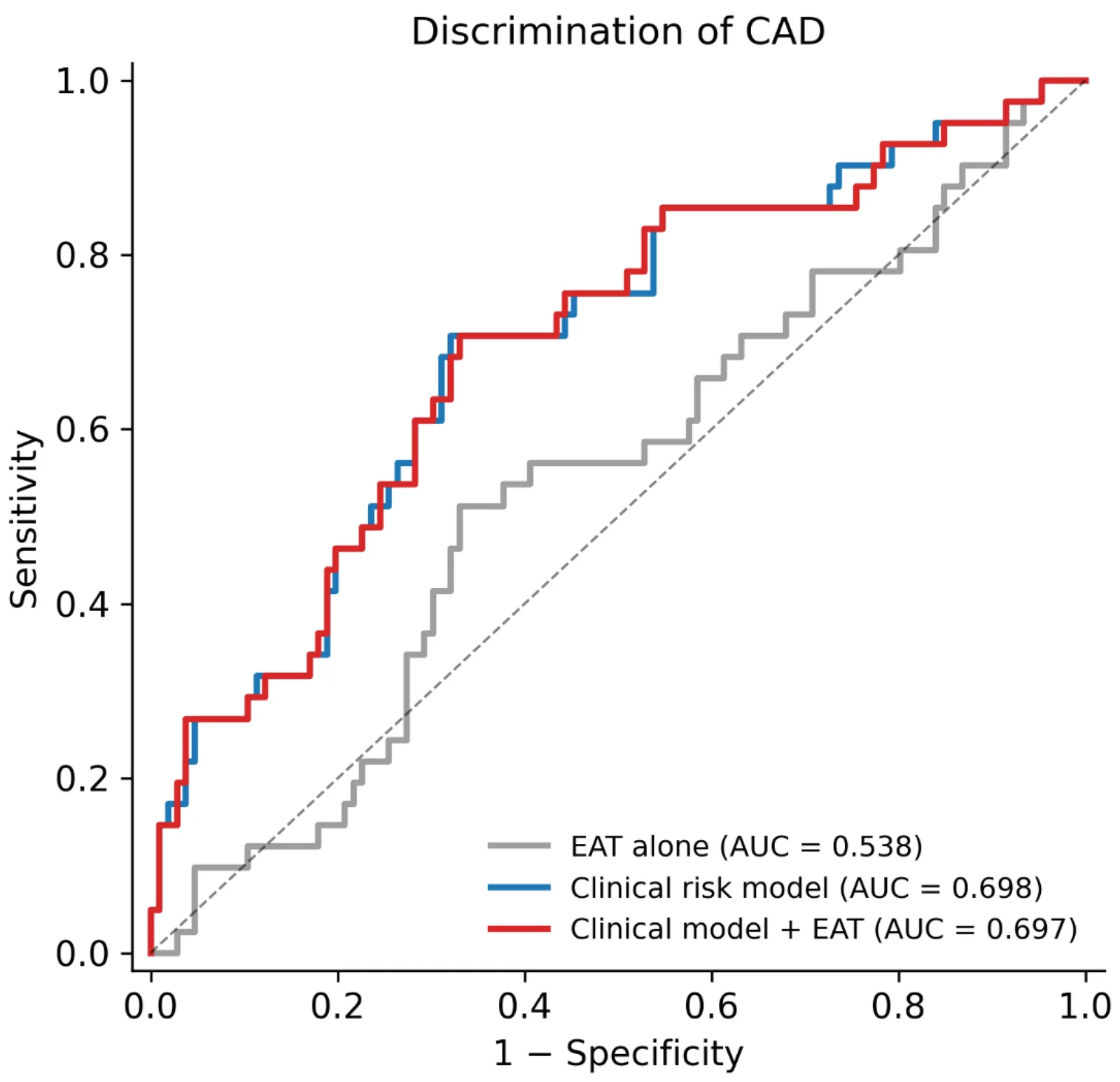
ROC curves for discriminating CAD. EAT volume alone (gray, AUC 0.538), the clinical risk model (blue; age, sex, BMI, HbA1c, diabetes; AUC 0.698), and the clinical risk model plus EAT volume (red, AUC 0.697). AUC, area under the curve; CAD, coronary artery disease; EAT, epicardial adipose tissue.

We further assessed the discriminative and incremental predictive value of EAT volume for CAD status (Table 3). EAT alone showed poor discrimination for CAD status, with an AUC of 0.538 (95% CI: 0.431–0.640). The clinical risk model, which included age, sex, BMI, HbA1c, and diabetes status, showed moderate discriminative ability, with an AUC of 0.698 (95% CI: 0.599–0.791). After adding EAT to the clinical risk model, the AUC did not improve, changing slightly to 0.697 (95% CI: 0.597–0.790) (Figure 1). The likelihood ratio testing showed no significant improvement in model fit after the addition of EAT (*p* = 0.945).

### 3.4. CatBoost Model Performance

**Figure 2:**
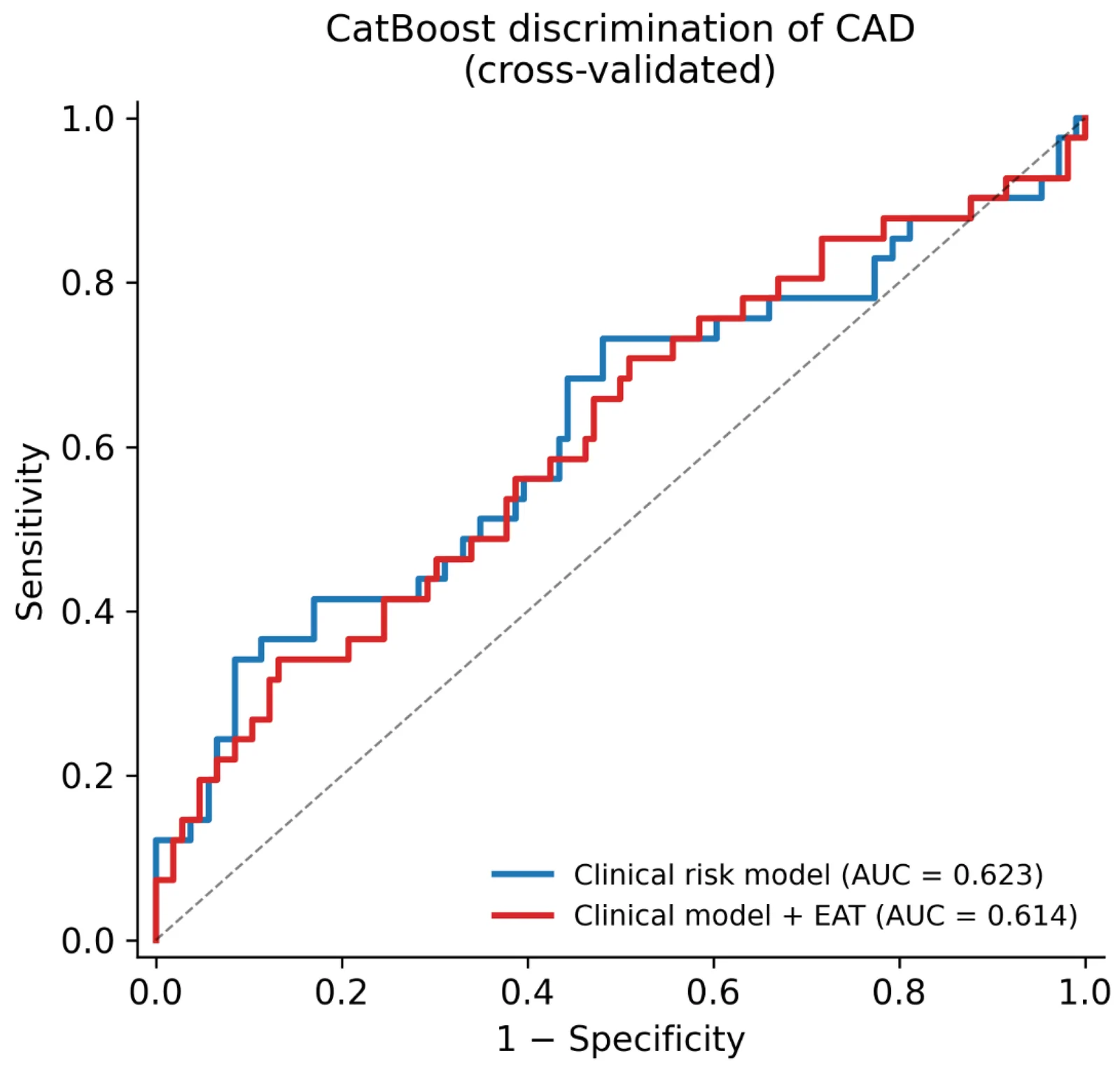
Cross-validated ROC curves from the CatBoost model for discriminating coronary artery disease. CAD, coronary artery disease.

To examine whether the absence of incremental value of EAT was specific to the logistic framework, we repeated the analysis using CatBoost. Using the same clinical feature set and repeated stratified five-fold cross-validation, the CatBoost clinical model achieved a cross-validated AUC of 0.623. Across cross-validation repetitions the AUC of the clinical model varied by a standard deviation of 0.02. Adding EAT volume did not improve discrimination (cross-validated AUC 0.614) (Figure 2).

### 3.5. SHAP Analysis

**Figure 3:**
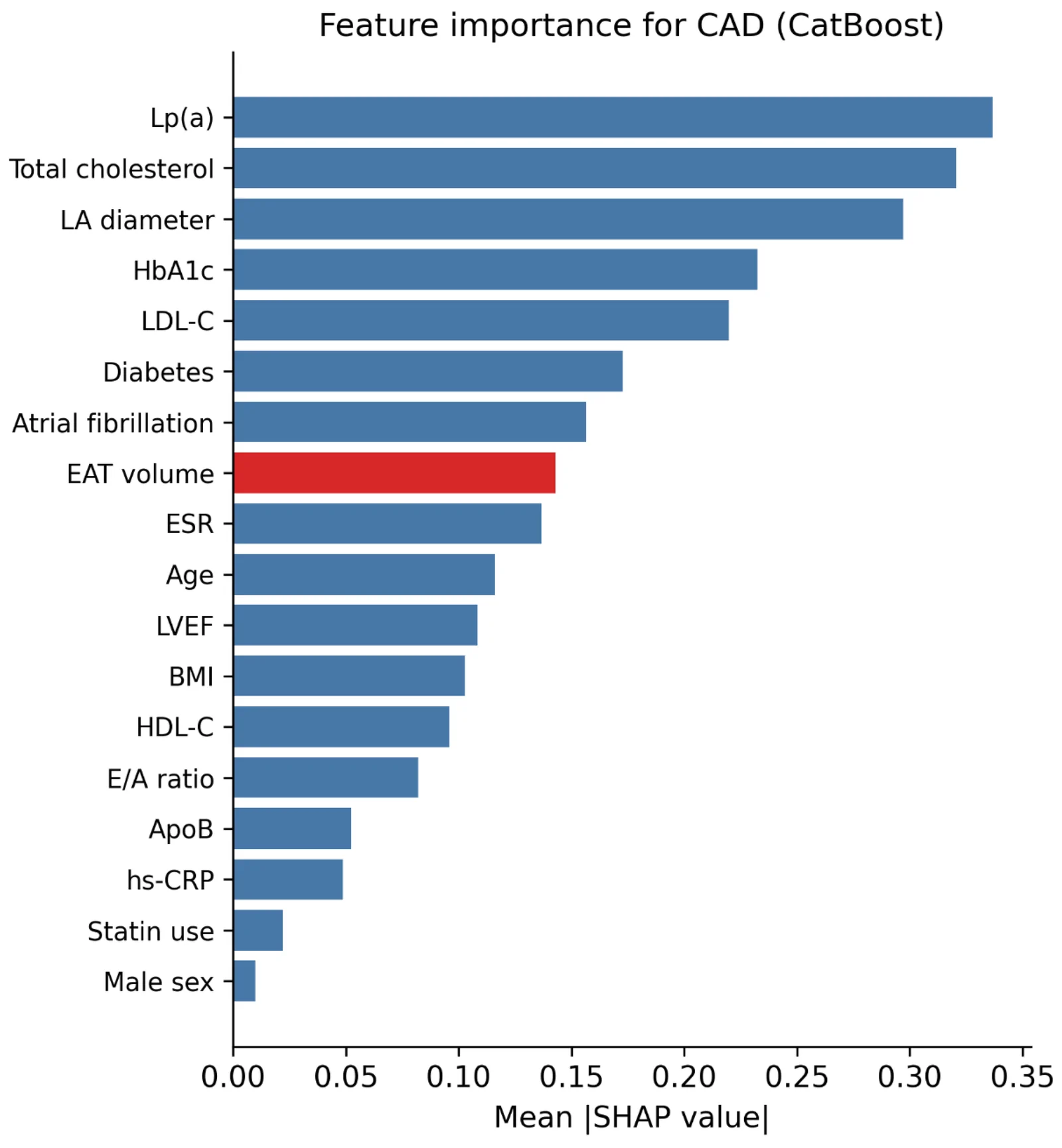
SHAP feature-importance ranking (mean |SHAP value|) from the CatBoost model incorporating clinical, laboratory, and echocardiographic variables. SHAP, SHapley Additive exPlanations.

To characterize the relative contribution of EAT volume within a multivariable machine-learning framework, we trained a CatBoost model incorporating clinical, laboratory, and echocardiographic variables and computed SHapley Additive exPlanations (SHAP). The features contributing most to CAD classification were lipoprotein(a), total cholesterol, left atrial diameter, HbA1c, and LDL-C (Figure 3). EAT volume ranked eighth of the eighteen features, with a mean absolute SHAP value (0.14) less than half that of the most influential feature (0.34), and lower than all major lipid, glycemic, and left atrial parameters. The events-per-variable ratio was 7.5 for the clinical model and 2.5 for the model including all clinical, laboratory, and echocardiographic variables.

## 4. Discussion

In this retrospective cohort of adults aged 80 years and older who underwent coronary imaging evaluation, CT-derived EAT volume showed little value for discriminating CAD. EAT volume was similar between patients with and without CAD, and it remained unassociated with CAD after adjustment for age, sex, BMI, diabetes, and HbA1c. The result was unchanged when the CAD group was limited to angiographic stenosis or to prior revascularization. Adding EAT to the clinical model did not improve discrimination, with the AUC changing from 0.698 to 0.697 and no significant improvement in model fit. The same pattern was observed in the CatBoost analysis, where inclusion of EAT failed to improve cross-validated performance. In the SHAP analysis, EAT ranked only eighth among the evaluated predictors, below lipoprotein(a), total cholesterol, left atrial diameter, HbA1c, LDL-C, diabetes, and atrial fibrillation. The null result held regardless of how we modeled the data. A nonlinear gradient-boosting model showed no incremental value of EAT, and SHAP ranked it below most metabolic, lipid, and echocardiographic variables.

The sample could detect an odds ratio of approximately 1.80, and weaker associations cannot be excluded, although an effect of that magnitude would be of uncertain clinical relevance in this population. Overall discrimination was modest, with a cross-validated AUC of 0.623 for the clinical model. The SHAP values therefore rank features within a model of limited accuracy, and with the low events-per-variable ratio these rankings should be considered exploratory. Precision was therefore limited, but the null result was consistent across all three analyses.

Our findings contrast with earlier studies, mostly in middle-aged cohorts, that found larger EAT volume in patients with CAD and a graded relationship with stenosis severity [5,16,22,23,24]. In pooled data from those cohorts, EAT volume was higher in patients with CAD by a standardized mean difference of 0.50 (95% CI 0.41 to 0.59) [5], and the adjusted odds ratio for obstructive stenosis was 1.055 (95% CI 1.033 to 1.078) per 10 cm^3^ [16]. In our cohort the two groups differed by 12 cm^3^ with almost complete overlap, and adding EAT to the clinical model left discrimination unchanged. Several factors may account for the lack of association in this population. First, EAT volume may lose specificity for CAD in advanced age. EAT is not a passive fat depot; it has local metabolic, inflammatory, and paracrine activity and lies in direct contact with the coronary arteries and myocardium [1,25,26]. Aging, however, brings chronic low-grade inflammation, shifts in adipose tissue phenotype, and a heavy burden of cardiometabolic comorbidity, against which EAT accumulation may become common in patients with and without CAD alike. Consistent with this, median EAT volume was similar and widely overlapping between the two groups in our cohort. Such a measurement may reflect generalized age-related visceral adiposity rather than a CAD-specific marker. Second, the clinical meaning of traditional cardiovascular risk factors shifts in very old adults, and EAT may behave similarly. In our cohort, patients with CAD had lower total cholesterol and LDL-C than those without CAD, and statin use was more frequent among them. This pattern is plausible in a population with established or suspected vascular disease. Similar attenuation or reversal of conventional risk-factor associations can all distort the usual association between lipid levels and disease, and these finding have been reported in geriatric populations [12,27,28,29]. Third, both survival and treatment may have weakened the difference in EAT between groups. Individuals with a high burden of inflamed epicardial fat and early, severe coronary disease are less likely to reach very old age. Survivors are enriched for those who carry substantial EAT without overt coronary consequences, a competing-risk selection that may underlie the reverse epidemiology described in geriatric cohorts [12]. Treatment could also act in the same direction. Patients with CAD in our cohort more often received statins (66.7% vs. 48.2%), and statin therapy reduces both EAT volume and its inflammatory activity, including EAT attenuation on CT [30,31]. Our own data, however, do not support this second pathway. Adding statin use to the fully adjusted model left the association between EAT and CAD unchanged, whereas statin use was itself positively associated with CAD and slightly improved discrimination. Survival selection, which cannot be tested in a cross-sectional cohort, remains the more likely of the two.

Non-contrast CT-derived EAT volume is attractive because it is simple, non-invasive, and can be obtained opportunistically from existing imaging. Nevertheless, in adults aged ≥80 years, EAT volume should not be used as a stand-alone marker to raise or lower suspicion of CAD. Clinical assessment in this age group still needs to rely on symptoms, functional status, diabetes, atrial fibrillation, lipoprotein(a), and the overall risk–benefit profile of further coronary testing. A low EAT volume should not reassure clinicians that CAD is absent, and a high EAT volume alone should not be taken as evidence of CAD.

Several limitations should be noted. First, this was a single-center retrospective study with a modest sample size and relatively few CAD cases, which limited model complexity and the precision of the estimates. Second, inclusion required complete coronary imaging, echocardiographic, and laboratory data, which may have introduced selection bias and limited the generalizability of our findings. These criteria may have selected patients with a higher clinical likelihood of CAD and a wider range of EAT values, potentially increasing the likelihood of detecting an association. Nevertheless, EAT values remained substantially overlapping between patients with and without CAD. Selection bias may therefore not fully account for the null finding, although validation in consecutive, multicenter prospective cohorts is required. Third, the cohort consisted predominantly of men. Our findings should not be generalized to women, in whom EAT volume and its relationship with coronary disease may differ [32]. Forth, only EAT volume was assessed. EAT attenuation and pericoronary fat attenuation reflect adipocyte lipid and water content rather than depot size, and are considered more direct markers of adipose inflammation [33]. In very elderly patients with diffuse visceral adiposity, they may retain discriminative value that volume does not. Mean attenuation was not recorded during the original segmentation, and pericoronary attenuation requires coronary CT angiography, which was not uniformly available in this cohort. Our findings therefore apply to EAT volume and should not be extended to other measures of epicardial fat. A further limitation is that CAD was analyzed as a binary outcome; the full range of coronary atherosclerosis, from non-obstructive plaque to graded stenosis severity, was not explored. Epicardial fat volume has, however, also failed to correlate with continuous measures of stenosis severity such as the Gensini score in younger cohorts [34].

## 5. Conclusions

In adults aged 80 years and older, CT-derived EAT volume was not associated with CAD and added no discriminative value beyond conventional clinical risk factors. This null result was consistent across logistic regression, a nonlinear machine-learning model, and SHAP-based interpretation. Whether qualitative CT features of epicardial fat, such as attenuation, retain discriminative value remains an open question that warrants further study in independent very-elderly cohorts.

## Figures and Tables

**Table 1:** Baseline clinical characteristics stratified by CAD status.

Characteristics	Overall (*n* = 155)	CAD (*n* = 45)	Non-CAD (*n* = 110)	*p*-Value
***Demographics and Anthropometrics***				
Age (years)	86.5 ± 5.2	85.8 ± 5.0	86.7 ± 5.3	0.343
Male sex, n (%)	138 (89.0)	43 (95.6)	95 (86.4)	0.168
BMI (kg/m^2^)	25.0 ± 3.4	25.9 ± 3.6	24.6 ± 3.3	0.052
Systolic BP (mmHg)	140 ± 18	139 ± 18	140 ± 17	0.675
Diastolic BP (mmHg)	70 ± 10	69 ± 11	71 ± 10	0.294
Hypertension, n (%)	126 (81.3)	38 (84.4)	88 (80.0)	0.488
Diabetes mellitus, n (%)	60 (38.7)	25 (55.6)	35 (31.8)	**0.010**
Atrial fibrillation, n (%)	11 (7.1)	8 (17.8)	3 (2.7)	**0.003**
Fatty liver disease, n (%)	85 (54.8)	24 (53.3)	61 (55.5)	0.950
Thyroid dysfunction	34 (21.9)	12 (26.7)	22 (20.0)	0.443
Statin Use, n (%)	83 (53.5)	30 (66.7)	53 (48.2)	0.055
***Biochemical and Lipid Profiles***				
TC (mmol/L)	3.98 (3.41, 4.71)	3.63 (3.15, 4.01)	4.25 (3.55, 4.72)	**0.005**
LDL-C (mmol/L)	2.40 (1.94, 3.19)	2.10 (1.82, 2.45)	2.59 (1.98, 3.22)	**0.013**
HDL-C (mmol/L)	1.29 (1.12, 1.56)	1.20 (1.09, 1.49)	1.33 (1.13, 1.58)	0.090
TG (mmol/L)	1.12 (0.85, 1.68)	1.14 (0.83, 1.62)	1.12 (0.86, 1.71)	0.864
ApoA1 (g/L)	1.40 (1.29, 1.56)	1.35 (1.22, 1.58)	1.41 (1.31, 1.56)	0.073
ApoB (g/L)	0.80 (0.70, 1.00)	0.70 (0.60, 0.90)	0.80 (0.70, 1.00)	0.053
Lp(a) (mg/L)	92.00 (33.00, 215.50)	125.00 (40.00, 293.00)	66.50 (31.00, 186.75)	0.066
Free fatty acids (µmol/L)	496.00 (365.50, 631.50)	492.00 (348.00, 583.00)	497.50 (386.50, 648.50)	0.618
HbA1c (%)	5.90 (5.60, 6.60)	6.30 (5.80, 7.10)	5.80 (5.50, 6.30)	**0.002**
hsCRP (mg/L)	0.94 (0.43, 2.13)	1.08 (0.47, 2.67)	0.84 (0.40, 1.94)	0.289
ESR (mm/h)	10.00 (7.00, 17.50)	13.00 (8.00, 19.00)	9.50 (6.00, 15.00)	0.118
***Epicardial Adipose Tissue***				
EAT volume (cm^3^)	118.73 (76.72, 171.64)	129.75 (79.62, 163.22)	117.45 (76.72, 173.25)	0.716
***Echocardiographic Parameters***				
LA anteroposterior diameter (mm)	40 (36, 42)	41(39, 46)	39 (36, 41)	**0.001**
E/A ratio	0.9 (0.7, 1.0)	0.8 (0.6, 1.0)	0.9 (0.7, 1.1)	**0.046**
TRV (m/s)	2.2 (2.0, 2.4)	2.30 (2.1, 2.5)	2.20 (2.0, 2.4)	0.660
IVS thickness (mm)	9 (8, 10)	9 (8, 10)	9 (8, 10)	0.464
LVPW thickness (mm)	9 (8, 10)	9 (8, 10)	9 (8, 10)	0.720
LVEDD (mm)	47 (45, 50)	47 (45, 50)	48 (45, 50)	0.732
LVESD (mm)	29 (27, 31)	28 (27, 30)	29 (27, 31)	0.583
LVEF (%)	69.0 (66.0, 72.0)	68.5 (65.2, 73.0)	69.0 (66.0, 72.0)	0.946
LVFS (%)	38.9± 3.8	39.1 ± 4.1	38.8 ± 3.7	0.686

Normally distributed continuous variables are expressed as mean ± standard deviation, and non-normally distributed continuous variables are expressed as median (interquartile range). Categorical variables are expressed as counts and percentages. Bold *p*-values indicate statistical significance (*p* < 0.05). Abbreviations: ApoA1, apolipoprotein A1; ApoB, apolipoprotein B; BMI, body mass index; BP, blood pressure; CAD, coronary artery disease; EAT, epicardial adipose tissue; ESR, erythrocyte sedimentation rate; HbA1c, glycated hemoglobin A1c; HDL-C, high-density lipoprotein cholesterol; hsCRP, high-sensitivity C-reactive protein; IVS, interventricular septum; LA, left atrial; LDL-C, low-density lipoprotein cholesterol; Lp(a), lipoprotein(a); LVEDD, left ventricular end-diastolic diameter; LVEF, left ventricular ejection fraction; LVESD, left ventricular end-systolic diameter; LVFS, left ventricular fractional shortening; LVPW, left ventricular posterior wall; TC, total cholesterol; TG, triglyceride; TRV, tricuspid regurgitation velocity.

**Table 2:** Association between EAT volume and CAD across multivariable logistic regression models.

Variable	Model 1 OR (95% CI)	*p*-Value	Model 2 OR (95% CI)	*p*-Value	Model 3 OR (95% CI)	*p*-Value	Model 4 OR (95% CI)	*p*-Value
EAT volume, per 1-SD increase	1.06 (0.73–1.52)	0.766	0.93 (0.63–1.38)	0.721	0.98 (0.65–1.48)	0.930	1.00 (0.65–1.52)	0.988
Age, per year	0.97 (0.90–1.04)	0.400	0.97 (0.90–1.05)	0.480	0.98 (0.91–1.06)	0.685	0.99 (0.91–1.07)	0.741
Male sex	3.07 (0.66–14.21)	0.151	2.96 (0.63–13.86)	0.168	2.75 (0.57–13.15)	0.206	2.37 (0.49–11.55)	0.285
BMI, per 1-SD increase	-	-	1.43 (0.95–2.16)	0.087	1.30 (0.85–1.98)	0.233	1.30 (0.84–2.00)	0.236
Diabetes	-	-	-	-	2.15 (0.77–6.00)	0.145	2.36 (0.83–6.68)	0.107
HbA1c, per 1-SD increase	-	-	-	-	1.19 (0.73–1.92)	0.485	1.10 (0.67–1.80)	0.710
Statin use	-	-	-	-	-	-	2.04 (0.91–4.58)	0.083

Values are odds ratios and 95% confidence intervals. Odds ratios for EAT volume, BMI, and HbA1c are expressed per 1-standard-deviation increase. Model 1 was adjusted for age and sex. Model 2 was additionally adjusted for BMI. Model 3 was further adjusted for diabetes and HbA1c. Model 4 was further adjusted for statin use. Abbreviations: BMI, body mass index; CAD, coronary artery disease; CI, confidence interval; EAT, epicardial adipose tissue; HbA1c, glycated hemoglobin A1c; OR, odds ratio; SD, standard deviation.

**Table 3:** Discriminative and incremental predictive value of EAT for CAD.

Model	Predictors	AUC	95% CI	LRT *p*-Value
EAT volume alone	EAT volume	0.538	0.431–0.640	-
Clinical risk model	Age, sex, BMI, HbA1c, diabetes status	0.698	0.599–0.791	-
Clinical risk model + EAT	Age, sex, BMI, HbA1c, diabetes status, EAT volume	0.697	0.597–0.790	0.945

The likelihood-ratio test (LRT) compared the clinical risk model with and without EAT volume. AUC, area under the receiver operating characteristic curve; BMI, body mass index; CAD, coronary artery disease; CI, confidence interval; EAT, epicardial adipose tissue; HbA1c, glycated hemoglobin A1c; LRT, likelihood-ratio test.

## Data Availability

The data that support the findings of this study are available from the corresponding authors upon reasonable request.

## Supplementary Materials

The following supporting information can be downloaded at: https://www.mdpi.com/article/10.3390/jcdd13090424/s1, Table S1: Univariable logistic regression analysis of candidate predictors for CAD status, Table S2: Sensitivity analyses for the association between EAT volume and CAD status.
