# Supplementary material for: Epicardial Adipose Tissue Shows No Significant Incremental Value for Discriminating Coronary Artery Disease in Adults Aged 80 Years and Older: A Machine Learning Analysis
Source: J Cardiovasc Dev Dis. 2026 Sep 1;13(9):424. doi: 10.3390/jcdd13090424 (PMC13607141; doi:10.3390/jcdd13090424)
Supplement: Supplementary file 1 [file jcdd-13-00424-s001.zip › jcdd-4380288-supplementary.pdf]

Supplementary Tables

Supplementary Table S1. Univariable logistic regression analysis of candidate predictors for CAD status

Variable	OR (95% CI)	p value	Overall p value
LA anteroposterior diameter	1.154 (1.062–1.255)	<0.001	<0.001
Atrial fibrillation status	0.151 (0.044–0.520)	0.003	0.002
Diabetes status	3.000 (1.400–6.427)	0.005	0.016
HbA1c	1.701 (1.159–2.496)	0.007	0.005
TC	0.574 (0.376–0.876)	0.010	0.007
Creatinine	1.016 (1.004–1.029)	0.010	0.007
RV anteroposterior diameter	1.124 (1.024–1.233)	0.014	0.011
Cystatin C, per 1 mg/L increase	2.901 (1.120–7.514)	0.028	0.026
Urea, per 1 mmol/L increase	1.170 (1.016–1.348)	0.030	0.026
LDL-C, per 1 mmol/L increase	0.606 (0.383–0.958)	0.032	0.025
Statin use	2.151 (1.043–4.436)	0.038	0.035
BMI, per 1 kg/m² increase	1.116 (1.002–1.242)	0.046	0.042
E/A ratio	0.213 (0.047–0.973)	0.046	0.035
Waist circumference	1.040 (1.000–1.082)	0.049	0.044
Weight	1.030 (0.996–1.066)	0.080	0.075
ApoA1	0.241 (0.045–1.289)	0.096	0.086
Male sex	3.395 (0.743–15.502)	0.115	0.073
HDL-C	0.408 (0.128–1.298)	0.129	0.120
ApoB	0.293 (0.058–1.487)	0.138	0.128

Variable	OR (95% CI)	p value	Overall p value
Thyroid dysfunction	1.828 (0.816–4.095)	0.143	0.148
Diastolic BP	0.981 (0.947–1.016)	0.281	0.277
hs-CRP	1.110 (0.912–1.349)	0.298	0.303
ESR	1.016 (0.986–1.048)	0.302	0.306
IVS thickness	0.862 (0.637–1.167)	0.337	0.333
LVESD	0.954 (0.865–1.052)	0.344	0.341
LVEF	0.093 (0.001–13.868)	0.352	0.347
Age	0.968 (0.904–1.037)	0.353	0.347
Diabetes status	1.750 (0.535–5.727)	0.355	0.016
LVEDD	0.967 (0.881–1.061)	0.478	0.480
TG	0.850 (0.502–1.439)	0.544	0.537
Lp(a)	1.000 (0.999–1.002)	0.623	0.627
Systolic BP	0.996 (0.976–1.016)	0.665	0.664
Free fatty acids	1.000 (0.998–1.001)	0.667	0.665
LVFS	8.529 (0.000–168221.135)	0.671	0.671
TRV	1.216 (0.347–4.254)	0.760	0.761
EAT volume	1.001 (0.995–1.006)	0.798	0.798
Fatty liver disease	0.918 (0.458–1.841)	0.810	0.810
Height	1.004 (0.958–1.053)	0.862	0.862
LVPW thickness	0.977 (0.683–1.398)	0.898	0.898

Note: Odds ratios were calculated using univariable logistic regression models with CAD status as the dependent variable. CAD status was coded as CAD = yes versus non-CAD. For categorical variables, odds ratios compare the listed category with the reference category. Overall p values were calculated using likelihood ratio tests when applicable.

Abbreviations: ApoA1, apolipoprotein A1; ApoB, apolipoprotein B; BMI, body mass index; BP, blood pressure; CAD, coronary artery disease; CI, confidence interval; EAT, epicardial adipose tissue; ESR, erythrocyte sedimentation rate; HbA1c, glycated hemoglobin A1c; HDL-C, high-density lipoprotein cholesterol; hs-CRP, high-sensitivity C-reactive protein; IVS, interventricular septum; LA, left atrial; LDL-C, low-density lipoprotein cholesterol; Lp(a), lipoprotein(a); LVEDD, left ventricular end-diastolic diameter; LVEF, left ventricular ejection fraction; LVESD, left ventricular end-systolic diameter; LVFS, left ventricular fractional shortening; LVPW, left ventricular posterior wall; OR, odds ratio; RV, right ventricular; TC, total cholesterol; TG, triglyceride; TRV, tricuspid regurgitation velocity.

Supplementary Table S2. Sensitivity analyses for the association between EAT volume and CAD status

Model	Adjustment variables	EAT OR (95% CI)	p value
Primary model	Age, sex, BMI, diabetes status, HbA1c	0.982 (0.652–1.478)	0.930
Lipid sensitivity model 1	Primary model + LDL-C	0.992 (0.652–1.510)	0.971
Lipid sensitivity model 2	Primary model + LDL-C + statin use	0.994 (0.652–1.516)	0.978
Extended clinical sensitivity model	Primary model + LDL-C + atrial fibrillation + LA diameter + E/A ratio	0.810 (0.458–1.433)	0.469

Note: EAT odds ratios are expressed per 1-standard-deviation increase in EAT volume. The primary model included age, sex, BMI, diabetes status, and HbA1c. Lipid sensitivity models further adjusted for LDL-C with or without statin use. The extended clinical sensitivity model further included LDL-C, atrial fibrillation, left atrial anteroposterior diameter, and the E/A ratio and was interpreted cautiously because of the smaller complete-case sample and limited number of CAD events.

Abbreviations: BMI, body mass index; CAD, coronary artery disease; CI, confidence interval; EAT, epicardial adipose tissue; HbA1c, glycated hemoglobin A1c; LA, left atrial; LDL-C, low-density lipoprotein cholesterol; OR, odds ratio.
